# Natural Alkaloids and Heterocycles as G-Quadruplex Ligands and Potential Anticancer Agents

**DOI:** 10.3390/molecules23020493

**Published:** 2018-02-23

**Authors:** Tong Che, Yu-Qing Wang, Zhou-Li Huang, Jia-Heng Tan, Zhi-Shu Huang, Shuo-Bin Chen

**Affiliations:** School of Pharmaceutical Sciences, Sun Yat-sen University, Guangzhou 510006, China; chetong@mail2.sysu.edu.cn (T.C.); wangyuq2@mail2.sysu.edu.cn (Y.-Q.W.); huangzhli@mail2.sysu.edu.cn (Z.-L.H.); tanjiah@mail.sysu.edu.cn (J.-H.T.); ceshzs@mail.sysu.edu.cn (Z.-S.H.)

**Keywords:** natural alkaloids, anticancer agents, drug design, G-quadruplex

## Abstract

G-quadruplexes are four-stranded nucleic acid secondary structures that are formed in guanine-rich sequences. G-quadruplexes are widely distributed in functional regions of the human genome and transcriptome, such as human telomeres, oncogene promoter regions, replication initiation sites, and untranslated regions. Many G-quadruplex-forming sequences are found to be associated with cancer, and thus, these non-canonical nucleic acid structures are considered to be attractive molecular targets for cancer therapeutics with novel mechanisms of action. In this mini review, we summarize recent advances made by our lab in the study of G-quadruplex-targeted natural alkaloids and their derivatives toward the development of potential anticancer agents.

## 1. Introduction

Guanine-rich nucleic acid sequences are able to fold into four-stranded secondary structures known as G-quadruplexes, which arise from the self-association of guanine bases by Hoogsteen hydrogen bonding [1]. Putative quadruplex sequences (PQS) are widespread throughout the human genome, especially at telomeres and regulatory regions of oncogenes [2,3,4]. G-quadruplexes are now thought to play important roles in several biological events associated with cancer, particularly in telomere maintenance and the expression of oncogenes, and have received much attention [5,6,7,8]. The conformations of G-quadruplexes provide recognition sites for small molecules, and thus, these structures are promising targets for the design of anticancer drugs [9,10]. 

Although there remains a long way to go in the development of potent drugs that target G-quadruplexes, some promising lead compounds have been obtained. For example, telomestatin, which selectively binds to telomeric G-quadruplex DNA, was able to cause telomere dysfunction by inhibiting telomerase activity and disturbing the sheltering complex [11,12]. Pyridostatin (PDS) could inhibit the transcription of G-quadruplex-related genes while interacting with promoter G-quadruplexes [13]. Notably, the ribosomal DNA (rDNA) G-quadruplex ligand Quarfloxin (CX-3543), which was able to disrupt nucleolin/rDNA G-quadruplex complexes and block the biogenesis of rRNA, has once enter the phase II clinical trials [14]. There is large evidence that ligands which can stabilize G-quadruplex are able to interfere with the G-quadruplex related biological events associated with cancer [15]. 

Some of these reported G-quadruplex ligands are natural alkaloids [16,17], which are an important class of natural products that have diverse and significant biological activities, such as antimalarial and anticancer activities. Because these compounds exhibit chemical diversity and structural complexity and are available from natural sources, natural alkaloids and their derivatives, such as telomestatin, the quindoline derivative CX-3543, the bis-quinoline derivative PDS and the acridine derivative BRACO-19, have been investigated intensively for their potential as G-quadruplex ligands [18,19,20]. In this paper, we summarize the previously published research from our lab on G-quadruplex-targeting natural alkaloids and heterocycles as potential anticancer agents.

## 2. G-Quadruplex Structures

**Figure 1:**
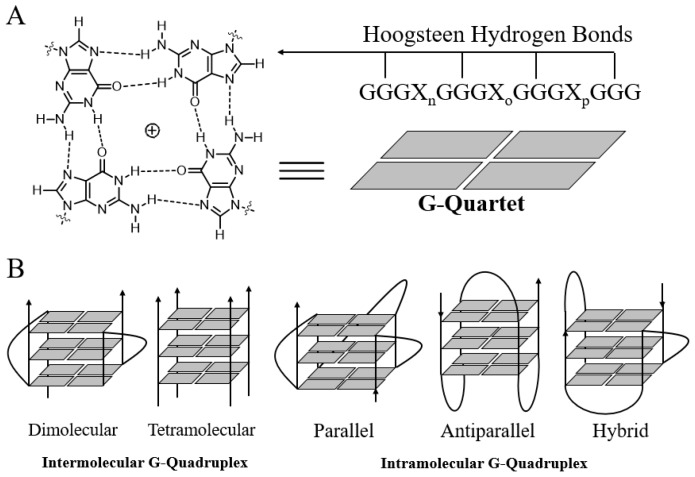
Structures of the G-quartet (**A**) and G-quadruplexes (**B**).

Guanine-rich strands have high propensity to self-associate into unusual structures called G-quadruplexes (Figure 1), which were first reported by Davies and co-workers in 1962 [21]. G-quadruplex topology is clearly different from that of B-DNA and other types of DNA. The basic building block of a G-quadruplex is a structure in which four guanine residues form a ring-like, aromatic, planar G-tetrad structure via Hoogsteen hydrogen bonds between the N1, N7, O6 and N2 sites. With the advent of X-ray crystallography, nuclear magnetic resonance spectroscopy (NMR) and other powerful technologies, the structures of many G-quadruplexes have been resolved [22,23]. G-quadruplex structures can fold due to either intra- or intermolecular interactions of G-rich strands. Structural polymorphism arises mostly from the properties of the loop, such as variations in strand stoichiometry, strand polarity, and glycosidic torsion angle and the sites on the loop that link to the guanine strand [24]. Moreover, the solution conditions, such as the presence of metal ions or ligands, or molecular crowding, may also influence the topology of quadruplexes. G-quadruplexes can form via intramolecular folding of a single G-rich sequence or by intermolecular association of two (dimeric) or four (tetrameric) separate strands. The relative arrangement of strand polarity can also give rise to structural polymorphism. For example, the polarities of the four strands in a G-quadruplex can be arranged as follows: all parallel strands; three parallel strands and one antiparallel strand; adjacent parallel strands; or alternating parallel strands. These arrangements lead to different conformations, namely, parallel, propeller, and antiparallel quadruplexes [25,26]. The conformations of G-quadruplexes formed from the folding of different G-rich sequences are distinct. G-quartet regions, loop regions, groove dimensions, and the negative electrostatic potential of the anionic backbone and the central channel of G-quadruplexes are critical elements that need to be considered in order to improve the binding selectivity of drug candidates [12,27,28,29].

## 3. Existence and Distribution of G-Quadruplexes

Computational predictions using simple algorithms suggest that over 370,000 sequence motifs (of the type G_≥3_N_1–7_G_≥3_N_1–7_G_≥3_N_1–7_G_≥3_) in the human genome have the potential to form G-quadruplex structures [2]. These PQSs are frequently found within human telomeric DNA, rDNA, transcription start sites, and promoter regions, suggesting that G-quadruplex structures may play a pivotal role in the regulation of a variety of cellular processes, including telomere maintenance, ribosome biogenesis, gene replication and transcription [3]. A method that combines G-quadruplex-dependent DNA polymerase stalling and next-generation sequencing (G4-seq) has been developed and was used to obtain a genome-wide map of G-quadruplex structures in purified, single-stranded human DNA. G4-seq has identified more than 700,000 G-quadruplexes in the human genome, which is much higher than the values predicted by earlier algorithms. A majority (70%) of these sequences either comprise G-tracts that are interrupted by non-guanine sequences and are predicted to form bulges or comprise extra-long loops [4]. Another method, which couples reverse transcriptase stalling with next-generation sequencing (rG4-seq), was developed and was used to characterize more than 13,000 G-quadruplexes in the human transcriptome. The enrichment of G-quadruplexes in transcripts that mediate RNA processing and stability reveals that RNA G-quadruplexes may play roles in the regulation of transcripts via miRNA-mediated gene regulation and alternative polyadenylation [7]. On the other hand, ~10,000 G-quadruplexes were identified in human chromatin by an antibody-based G-quadruplex chromatin immunoprecipitation and high-throughput sequencing approach (G4 ChIP-seq); these G-quadruplexes were identified predominantly in regulatory, nucleosome-depleted regions [5]. G-quadruplex structures are enriched in the promoters and 5′ UTRs of highly transcribed genes, particularly in genes related to cancer, and in somatic copy number alterations (SCNAs). The findings of G-quadruplex-related sequencing suggest that the variety and number of potential G-quadruplexes are greater than originally predicted.

Studies involving the visualization of G-quadruplexes by antibodies and small-molecule probes have provided crucial evidence of the existence of G-quadruplexes in cells [30]. Schaffitzel et al., who were the first to report the visualization of G-quadruplex formation in a biologically relevant context, used a G-quadruplex-selective antibody, Sty49, to show that G-quadruplexes form at telomeres in the macronuclei of the ciliate *Stylonychia lemnae* [31]. DNA G-quadruplexes were then observed at the ends of human chromosomes by using the radiolabeled ligand ^3^H-360A and the fluorescent ligand BMVC [32,33]. Subsequently, DNA G-quadruplexes were visualized in the nuclei of human cells by in situ fluorescence labelling of PDS and immunofluorescence microscopy using the G-quadruplex-specific antibody BG4 [13,34]. Notably, the number of BG4 foci increased after the exposure of live cells to G-quadruplex ligands, including PDS, Phen-DC3 and TMPyP4, which demonstrates that these ligands trap the G-quadruplex structures formed in cells. Furthermore, interactions between small molecules and DNA G-quadruplexes were visualized in live cells by fluorescence-lifetime imaging microscopy using DAOTA-M2. Several fluorescent probes capable of discriminating DNA G-quadruplexes from single- and double-stranded nucleic acids have also been described previously [35,36,37,38,39]. Compared with the detection of DNA quadruplexes, the detection of cellular RNA quadruplexes was more challenging. RNA quadruplexes were first detected by immunodetection using BG4 in 2014 [40]. This finding provides substantive evidence for the existence of RNA G-quadruplexes in human cells. On the other hand, this investigation also demonstrates that the ligand carboxyPDS, which binds to G-quadruplexes in vitro, can trap endogenous RNA G-quadruplexes when used in cells. This result corroborates the results obtained by using stabilizing ligands that target G-quadruplexes in a cellular context. RNA G-quadruplexes were then observed in live cells by using the multiphoton turn-on fluorescent probe NaphthoTASQ in 2015 [41]. More recently, a G-quadruplex-triggered fluorogenic hybridization (GTFH) probe reported by our research group specifically visualized the RNA G-quadruplex structure formed by the G-rich sequence within the 5′-UTR of NRAS mRNA in the cytoplasm of human cells [42]. Overall, antibodies and fluorescent probes capable of structure-specific detection of G-quadruplexes are powerful tools for basic biological research and for the exploration of G-quadruplexes as a potential drug targets.

## 4. G-Quadruplexes as Promising Targets for Anticancer Drug Design

### 4.1. Telomeric G-Quadruplexes 

Human telomeres comprise tandem repeats of the DNA motif TTAGGG together with associated telomeric proteins and feature a 50–300-nucleotide protrusions of single-stranded repeats from the 3′ end, termed the G-tail or G-overhang [43]. This G-overhang acts as a substrate for telomerase, a reverse transcriptase with an RNA component that is required for telomere extension. The structure and stability of telomeres are closely related to cancer, aging, and genetic stability. Due to the high frequency of TTAGGG repeats and due to the lack of competition from a complementary strand at the 3′ overhang, the formation of a G-quadruplex would be favored. 

The formation of a G-quadruplex can affect telomeres in profound ways, including (a) inhibiting telomerase activity by blocking the binding of telomerase to the single-stranded telomere substrate to elongate the telomeres. Telomerase is overexpressed in 80–85% of cancer cells and primary tumors, making it an attractive target for cancer therapeutics [44]; (b) telomere uncapping by dissociation of telomere-binding proteins (e.g., POT1, TRF1 and TRF2) from the end of the telomere. Telomere uncapping can lead to telomeric dysfunction, which is characterized by end-to-end fusion, inappropriate recombination, anaphase bridges, and G-overhang degradation, and leads to either apoptosis or senescence [45]; and (c) interfering with telomere replication by impairing replication fork progression. These findings prompted the development of several selective G-quadruplex-binding small molecules with the aim of developing novel telomere-based anticancer therapies. 

Recently, a number of studies showed that the 3′-terminal single-stranded overhang could form several consecutive quadruplex units connected by TTA linkers, called telomeric multimeric G-quadruplexes [46,47,48,49]. This distinctive structure enables the possibility of designing small molecules that are able to discriminate telomeric G-quadruplexes from a large number of other G-quadruplexes with various biological functions [50]. Thus, it has been suggested that small molecules that specifically target telomeric multimeric G-quadruplexes might be more promising as anticancer agents and might have fewer side effects. 

### 4.2. Oncogene Promoter Region G-Quadruplexes 

In addition to telomeric G-quadruplexes, several putative G-quadruplex sequences have been identified near transcription start sites and gene promoters, indicating the key role of these structures in the regulation of various genes. These gene promoters include the promoters of c-MYC, c-KIT, bcl-2, K-RAS, VEGF, RB, PDGF-A, hTERT [51,52,53,54,55,56,57,58]. More importantly, altered expression of these oncogenes are recognized as hallmarks of cancer. At the turn of the century, Hanahan and Weinberg proposed six vital cellular and microenvironmental processes that are aberrantly regulated during oncogenic transformation and malignancy [59]. When each of these categories is examined, one or more critical proteins are found to have G-quadruplexes in the core or proximal promoter regions. Transcriptional repression of oncogenes by stabilization of G-quadruplex structures or by inhibiting the binding of related proteins to these structures could be a novel anticancer strategy. 

c-MYC is one of the best-studied oncogenes. Up to 80% of all solid tumors, including gastrointestinal, ovarian and breast cancer tumors, overexpress c-MYC as do many non-Hodgkin’s lymphoma tumors [60]. The nuclease-hypersensitive element (NHE III_1_) in the c-MYC promoter regulates 85–90% of c-MYC transcription. The region contains 27 guanine-rich bases (referred to as Pu27 herein) that can fold into a G-quadruplex that is considered a transcription repressor element. Several G-quadruplex-binding ligands possess the ability to promote and/or stabilize the c-MYC G-quadruplex structure, thus repressing c-MYC transcription [51]. Generally, gene transcription is triggered and regulated by transcription factors. Transcription factors promote or repress gene transcription by recognizing and binding to certain regions in promoters and then recruiting other factors. In most cancer cells, the expression of transcription factors is usually abnormal, which theoretically makes these proteins promising targets for the development of anticancer drugs. 

### 4.3. RNA G-Quadruplexes

There is emerging evidence that 5′- and 3′-untranslated regions as well as open reading frames (ORFs) contain putative RNA G-quadruplexes. Due to the absence of a complementary strand in RNA molecules, it has been widely held that G-rich RNA sequences are more susceptible to the formation of G-quadruplex structures. Unlike the highly polymorphic DNA G-quadruplex structures that depend on the surrounding conditions, RNA G-quadruplexes almost exclusively adopt a single conformation. The presence of a 2′-hydroxyl group in the ribose sugar results in additional steric constraints on the G-quadruplex topology. These 2′-hydroxyl groups prevent the base from adopting a syn-conformation, instead strongly favoring the anti-conformation (by restraining the glycosidic torsion angle) and imposing additional constraints on sugar puckering (ribose has a preference for C3′-endo puckering). As a consequence, the topology of RNA G-quadruplexes is limited to the parallel conformation, in which all four strands are oriented in the same direction. There is ample evidence that RNA G-quadruplexes exist in the UTRs and ORFs of cancer-related genes that regulate transcription, alternative splicing and translation. In addition, the unique chemical properties of RNA G-quadruplexes suggest that it is possible to develop small molecules that selectively bind to RNA G-quadruplexes rather than DNA G-quadruplexes. This preferential binding is exemplified by the small molecule carboxyPDS, which exhibits high molecular specificity for RNA G-quadruplexes over DNA G-quadruplexes. Selective intervention by targeting only RNA G-quadruplexes may be a strategically important therapeutic approach for cancer. 

Recently, a high-throughput method was developed that identifies RNA G-quadruplex regions on the basis of the propensity of the RNA to stall reverse transcriptase in a K^+^-dependent manner. By applying this method to RNA from mammalian cell lines and yeast, >10,000 endogenous regions that form RNA G-quadruplexes in vitro were identified, thereby expanding the catalog of endogenous regions with experimentally confirmed propensity to fold into RNA G-quadruplex structures by a factor of >100. To determine the folding state of these RNA G-quadruplex regions in vitro and in cells, dimethyl sulfate (DMS) treatment was performed before mapping the reverse transcriptase stops. The results showed that, in contrast to previous assumptions, regions that folded into RNA G-quadruplex structures in vitro were overwhelmingly unfolded in eukaryotic cells [61]. Taken together, various independent studies on RNA G-quadruplexes reveal promising regulatory functions of these structures in multiple biological processes. However, these challenging puzzles have only been partially solved, and comprehensive studies should be undertaken to understand the behavior of RNA G-quadruplexes, such as the folding dynamics, stability and biological relevance in vivo and the detailed mechanism of RNA G-quadruplexes involved in regulatory processes. 

## 5. Natural Alkaloids and Their Derivatives as G-Quadruplex Ligands

### 5.1. Methods to Discover G-Quadruplex Ligands

As mentioned above, G-quadruplexes play important roles in several biological events associated with cancer. Selective intervention by targeting G-quadruplexes may be a strategically important therapeutic approach for cancer. Thus, the discovery of potential G-quadruplex was important. Generally, a good G4 ligand binds G-quadruplex with strong potency and selectivity over duplex and other nucleic acid structures in vitro. While in vivo, they are potent to interfere G4-involved biological events, such as the telomere maintenance and gene expression. Various methods, including those in vitro, in cells and in silicon, have been developed and applied in the screen of G-quadruplex ligands. Several frequently used G-quadruplex ligand screening methods are summarized in Table 1. Besides, optimization based on these known G-quadruplex interactive moieties is emerging as an effective approach to develop new G-quadruplex ligands, which will be elaborated in the following parts.

### 5.2. Quindoline Derivatives

**Figure 2:**
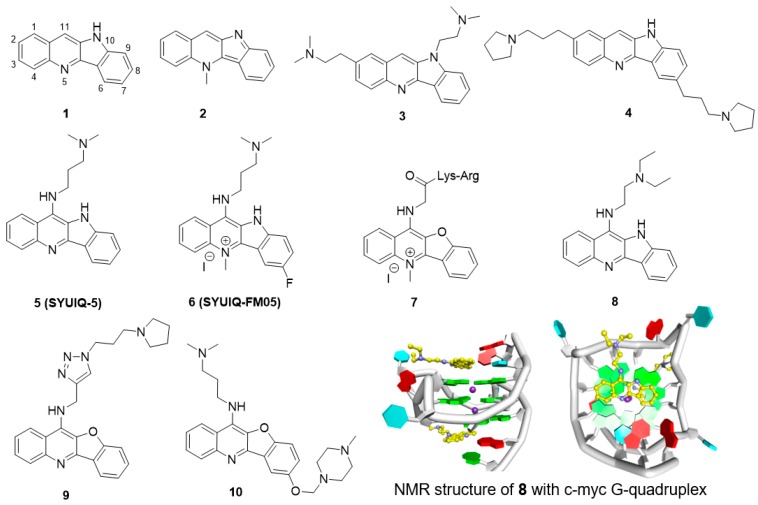
Structures of quindoline derivatives and binding mode with G-quadruplex.

Quindoline (**1**, Figure 2) is a naturally occurring indolo[3,2-*b*]quinoline alkaloid. Quindoline was originally synthesized in 1906 and has since been prepared from cryptolepine (**2**). Quindoline was first extracted from the African plant *Cryptolepis sanguinolenta* (Periplocaceae) in 1978. This compound has antimicrobial, antimalarial, anti-inflammatory, and antihypolipidemic activities. In 2000, Neidle et al. first reported that the 2,10-disubstituted quindoline derivative **3** could bind to G-quadruplex structures in human telomeres, showing it to have modest cytotoxicity against several cancer cells as well as inhibitory activity against telomerase, with an IC_50_ (^tel^IC_50_) of 16 μM [69]. They further determined that the 2,7-disubstituted quindoline derivative **4** showed more potent activity than **3**, with a ^tel^IC_50_ value of 6.3 μM [70]. 

The 11-substituted quindoline derivatives designed and synthesized by our research group exhibited significantly improved ability to stabilize telomeric G-quadruplexes [71,72]. The introduction of electron donating groups at position 11 enhances the basicity of the nitrogen atom in the pyridine ring, thus enhancing the electrostatic interaction between the derivatives and the negative electrostatic center of the G-quadruplex. The most active derivative, **5** (SYUIQ-5), showed improved inhibition of telomerase activity (^tel^IC_50_ = 0.44 μM) and induced a marked cessation in cell growth and a cellular senescence phenotype in human leukemia K562 cells and colon cancer SW620 cells [73]. The growth cessation was accompanied by a shortening of telomere length and upregulation of the expression of the cyclin-dependent kinase inhibitors p16, p21 and p27.

Further studies on SYUIQ-5 indicated that the 11-alkylamino group on quindoline could lead to in situ protonation of the 5-*N* atom [74]. Molecular modeling studies revealed that the crescent-shaped aromatic core was stacked on two guanine residues of the G-quartet, and the 5-*N* electropositive center overlapped with the cation channel of the quadruplex. However, the pKa values of the 5-*N* atoms of the 11-aminoquindolines were 8.2–8.4 and thus could be easily influenced by the conditions in the solution. An alternative to the introduction of a positive charge via in situ protonation was *N*-methylation at the 5-position. Based on this hypothesis, a series of 5-*N*-methyl quindoline derivatives were designed for screening ligands with better binding ability and that selectively recognized telomeric G-quadruplexes. The results showed that the 5-*N*-methyl quindoline derivatives not only exhibited significantly higher binding affinity for G-quadruplexes (approximately 5-fold higher than the non-methylated derivatives) but also exhibited selectivity toward antiparallel telomeric quadruplexes. Derivative **6** markedly inhibited telomerase activity in a cell-free system (^tel^IC_50_ = 0.31 μM). The determination of short-term cell viability by a two-day cytotoxicity assay (MTT assay) showed that **6** had a potent inhibitory effect on leukemia HL60 cells and lymphoma CA46 cells. Long-term exposure of HL60 and CA46 cells to **6** induced marked cessation of population growth, a cellular senescence phenotype, and shortening of telomere length.

Quindoline derivatives were capable of interacting with the telomeric G-quadruplex structure and exhibited inhibition of telomerase activity. However, studies showed that cryptolepine could interact with the CC sites of the DNA fragment d(CCTAGG)_2_ in a base-stacking intercalation mode. Thus, further studies were undertaken to improve the selectivity of these compounds. It has been reported that a combination of peptides that selectively bind to DNA scaffolds would greatly increase the selectivity of these peptides for G-quadruplexes versus duplex DNA. A series of peptidyl-benzofuroquinoline conjugates were designed and synthesized. The positive charge and alkyl-chain length of the dipeptidyl side chain proved to be important for the interaction of the side chain with the G-quadruplex. Derivative **7**, with a -Lys-Arg dipeptide fragment, exhibited the most potent telomerase inhibition (^tel^IC_50_ = 5.5 μM) and had 50 times higher selectivity for telomeric G-quadruplex than for the duplex. Additionally, cellular experiments also showed a marked cessation in population growth and a cellular senescence phenotype as well as shortening of the telomere length [75].

In addition to telomeric single-stranded overhangs, G-quadruplexes are also present in the promoter or regulatory regions of important oncogenes such as c-MYC, BCL-2 and c-KIT. c-MYC is an important proto-oncogene, and the aberrant overexpression of this gene is always associated with a variety of malignant tumors. Further studies have shown that SYUIQ-5 can also interact with the c-MYC G-quadruplex and inhibit the expression of this gene. A PCR stop assay showed that in the presence of SYUIQ-5, the Pu22 c-MYC oligomer was induced to form a G-quadruplex, which prevented hybridization with a complementary strand that overlapped with the last G repeat. The expression of c-MYC was seen to be markedly inhibited when the mRNA and protein levels in HL-60 and K562 cells were measured. Derivative **8**, with a short side chain, showed more potential stabilization of the c-MYC G-quadruplex. Further studies showed that **8** could inhibit the expression of c-MYC in the hepatocellular carcinoma cell line Hep G2. The binding of derivative **8** with c-MYC G-quadruplex was explored by Yang et al. [76]. Structural NMR results showed quindoline could stack effectively on both sides of G-quadruplex, and unexpectedly, the structure showed that the quindoline-induced reorientation of the flanking sequences led to the formation of a new binding pocket (Figure 2).

The quindoline derivatives were seen to interact with the G-quadruplex in the c-MYC gene with a certain degree of selectivity, and the specificities were further improved. The heteroaromatic 1,4-substituted 1,2,3-triazole ring system has attracted extensive interest in drug design owing to the wide applicability of this system as a synthetic building block endowed with pharmacological potential. A series of novel triazole-containing benzofuroquinoline derivatives (T-BFQs) were designed and synthesized by 1,3-dipolar cycloaddition of a series of alkyne and azide building blocks [77]. The selectivity of these novel T-BFQs toward c-MYC G-quadruplex DNA was significantly improved, and a distinct increase in binding affinity was observed. Further cellular experiments showed that derivative **9** specifically downregulated c-MYC gene transcription and expression in RAJI cells. Derivative **9** exhibited inhibition of tumor cell proliferation but had a weak effect on primary cultured mouse mesangial cells (in which proliferation does not depend on c-MYC expression). These results indicated that inhibition by **9** presumably occurs via the downregulation of transcription of the c-MYC gene. Tumor-bearing mice treated with derivative **9** had an average tumor volume of <400 mm^3^ and exhibited an inhibition rate of 38.1% (Table 2). 

Recently, four series of 7,11-disubstituted quindoline derivatives were also synthesized by introducing a second cationic amino side chain and a 5-*N*-methyl group as ligands to bind to the c-MYC promoter G-quadruplex. In vitro evaluations showed that the derivates exhibited increased stabilities and binding affinities, and most of the derivatives had better selectivity for the c-MYC G-quadruplex (over duplex DNA) compared to SYUIQ-5. Moreover, the new derivatives prevented NM23-H2, a transcription factor, from effectively binding to the c-MYC G-quadruplex. Further studies showed that derivative **10** downregulated c-MYC transcription by targeting the promoter G-quadruplex and disrupting the NM23-H2/c-MYC interaction in RAJI cells. Derivative **10** could inhibit Burkitt’s lymphoma cell proliferation through cell cycle arrest and apoptosis and suppress tumor growth in a human Burkitt’s lymphoma xenograft with an inhibition rate of 27.4% (Table 2) [78].

### 5.3. Isaindigotone Derivatives

**Figure 3:**
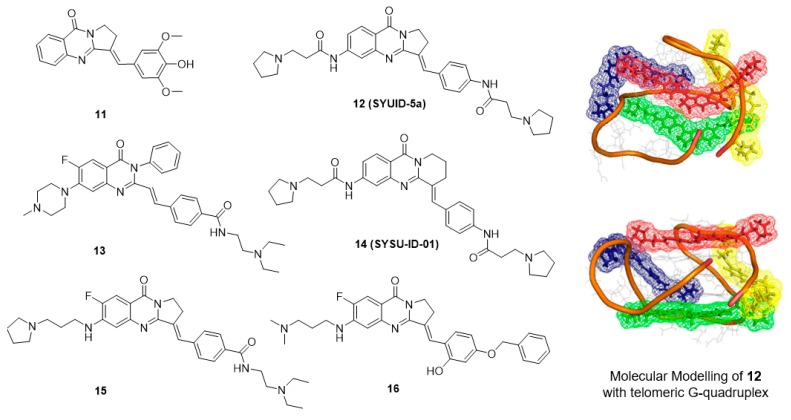
Structures of isaindigotone derivatives and binding mode with G-quadruplex.

Isaindigotone (**11**, Figure 3) is an alkaloid isolated from the root of the traditional Chinese herb *Isatis indigotica* Fort [80]. This compound exhibits excellent effects against influenza, epidemic hepatitis, and epidemic encephalitis. The structure of isaindigotone comprises a pyrrolo[2,1-*b*]quinazoline moiety conjugated with a benzylidene group. Our group first synthesized a series of isaindigotone derivatives to explore the effects of the derivatives on G-quadruplex stability and selectivity [81,82,83]. The unfused aromatic rings and tethered side chains of these derivatives seemed to allow the compounds to adopt flexible and adaptive conformations, which help in the recognition of telomeric G-quadruplex cores and grooves in a 4:1 binding mode. Derivative **12** significantly induced cell senescence and telomere shortening of HL-60 cells and CA46 cells at micromolar concentrations. A TRAP-LIG assay showed that derivative **12** inhibited telomerase activity with an ^tel^IC_50_ value of 7.8 μM. To better investigate how the planarity of an unfused aromatic ligand impacts the quadruplex-binding properties of the ligand, another series of isaindigotone derivatives were designed and were reported to stabilize the c-KIT G-quadruplex. The best derivative, **13**, reduced the transcription of c-KIT by 52.5% and exhibited significant cytotoxicity in a gastrointestinal stromal tumor cell line. SAR analysis showed that expansion of the aromatic system via the introduction of a benzene ring, a benzylidene group and two cationic amino side chains into the quinazolone moiety not only maximizes the stacking interactions of the derivatives with the G-quartet but also incorporates features of flexibility that prevent the derivative from intercalating with duplex DNA.

Currently, a number of small molecule ligands that stabilize G-quadruplexes have been developed [84,85]. However, selectively targeting a specific G-quadruplex is still a difficult task [86,87]. Typically, the interaction between protein and DNA is highly specific and stable. By targeting the interaction of a G-quadruplex-related protein with the G-quadruplex, new opportunities are emerging for the development of small molecules that target G-quadruplexes. In the process of c-MYC transcription, one of the transcription factors, NM23-H2, has an important role in transcriptional reactivation. Studies have revealed that NM23-H2 can recognize and bind to the c-MYC NHE III_1_ region and act as an unwinding protein on the G-quadruplex [88]. Therefore, the interaction of NM23-H2 with the c-MYC G-quadruplex might be a key process in the transcriptional regulation of the c-MYC gene, and derivatives that can interfere with the binding of NM23-H2 to the c-MYC G-quadruplex are expected to repress c-MYC transcription. An in-house small-molecule library screening by SPR identified the isaindigotone derivative **14** (SYSU-ID-01), which significantly interfered with the binding of NM23-H2 to the c-MYC promoter DNA [79]. Further analyses of the derivative-protein interaction and the protein-DNA interaction provided insight into the mode of action of SYSU-ID-01. Results of cellular evaluation showed that SYSU-ID-01 could abrogate the binding of NM23-H2 to the c-MYC promoter, resulting in downregulation of c-MYC transcription and dramatically suppressed HeLa cell growth. However, in primary cultured mouse mesangial cells, in which proliferation does not depend on c-MYC expression, instead of proliferation arrest, SYSU-ID-01 promoted cell proliferation. 

These results inspired further modification, and a series of new isaindigotone derivatives were designed and synthesized [89,90]. The abilities of these derivatives to interact with G-quadruplexes or with NM23-H2 and to disrupt the G-quadruplex-NM23-H2 interaction were evaluated. Among these derivatives, derivative **15** showed marked disruption of the G-quadruplex-NM23-H2 interaction. This compound exhibited significant effects on c-MYC-related processes in SiHa cells, including inhibition of transcription and translation, inhibition of cell proliferation, induction of apoptosis, and regulation of the cell cycle. In contrast, derivative **15** promoted cellular growth instead of proliferation arrest in primary cultured mouse mesangial cells. A SiHa xenograft mouse model of human cervical squamous cancer showed that after 3 weeks of treatment, compared with the vehicle group, treatment with derivative **15** at 20 mg/kg resulted in a statistically significant reduction in tumor weight, with a tumor growth inhibition rate of 35.2% (Table 2). Furthermore, to design and synthesize highly specific NM23-H2-binding ligands, the G-quadruplex-ligand features of the derivatives were removed, and only one positive amino side chain remained at the 6-position. A series of novel isaindigotone derivatives with a variety of substituents, such as alkyl, hydroxyl, alkoxy, alkamino, fluoro, and trifluoromethyl groups, at the 4′-position of the styrene ring were designed and synthesized and screened for NM23-H2-selective binding of ligands. Among these derivatives, derivative **16** exhibited significant binding affinity toward NM23-H2 and disrupted the NM23-H2/c-MYC G-quadruplex interaction in vitro and in cells. Inhibition of the transcription of the oncogene c-MYC by **16** induced cell-cycle G0/G1-phase arrest, cellular apoptosis, proliferation inhibition, and migration inhibition. In addition, **16** exhibited potent antiproliferation activity in several cancer cell lines and exhibited good antitumor activity by inhibiting the growth of cervical squamous cancer in BALB/C-nu/nu mice with a SiHa xenograft, with a tumor growth inhibition rate of 64.8% (Table 2).

### 5.4. Berberine Derivatives

**Figure 4:**
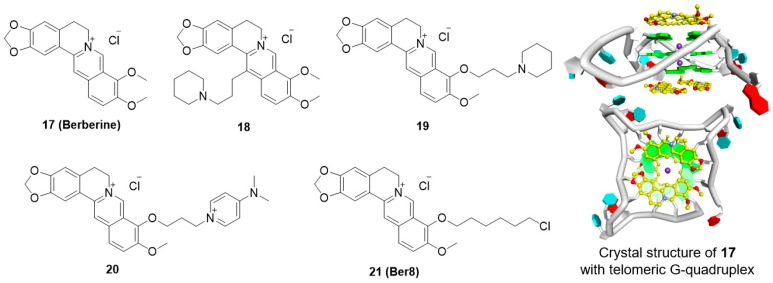
Structures of berberine derivatives and binding mode with G-quadruplex.

Berberine (**17**) is an alkaloid isolated from Chinese herbs that has long been used as an antimicrobial agent, and this compound has attracted a lot of attention for its antineoplastic effects. Berberine appears to suppress the growth of a variety of tumor cells, including breast cancer, leukemia, prostate carcinoma, and gastric carcinoma cells. In 1999, Tsuruo and coworkers were the first to report that berberine inhibits telomerase activity, thus interrupting telomere elongation [91]. The 13-substituted berberine derivative, **18,** then shows weak binding with telomeric G-quadruplex DNA and poor telomerase inhibition, while 9-*O*-subtituted berberine derivatives, by introducing a side chain with terminal amino group outside of the crescent-shaped ring system, show significantly improved ability to stabilize telomeric G-quadruplexes [92,93,94]. A recent X-ray structure of provided an important clue for interpreting data regarding the binding of berberine with telomeric G-quadruplex. The result showed that two berberine molecules could simultaneously interacting with each external G-tetrad via π-stacking, while the crescent-shaped ring of berberine stack over two guanines (Figure 4) [95]. 

Among these derivatives, derivative **19** markedly inhibited telomerase activity, with a ^tel^IC_50_ value of 16 μM. Introduction of a positively charged terminal aza-aromatic group may enhance the electrostatic interaction of the derivative with the phosphate backbone and improve the interaction with the loop region via π-π stacking. Derivative **20,** which had an aza-aromatic group, could selectively bind to telomeric G-quadruplexes over duplex DNA. Derivative **20** had a ^tel^IC_50_ value of 14 μM, which is much lower than that of berberine (^tel^IC_50_ = 70 μM). Recently, a newly synthesized berberine derivative, **21** (Ber8), with a chlorohexyl side chain at the 9-position, was seen to strongly interact with telomeric G-quadruplexes and could effectively induce acute cell growth arrest in cancer cells. 

The inhibition of cancer cell growth by Ber8 was associated with apparent cell cycle arrest, cell senescence, and profound DNA damage at telomeric regions. Further mechanical studies revealed that Ber8 could not only increase the formation of endogenous telomeric G-quadruplexes in cancer cells but also delocalize TRF1 and POT1 from the telomere and induce telomere uncapping. In vivo experiments showed that **21** exhibited tumor-inhibition activity in SiHa xenograft tumors (Table 2) [96,97].

### 5.5. Other Alkaloid Derivatives

#### 5.5.1. Quinazoline Derivatives 

**Figure 5:**
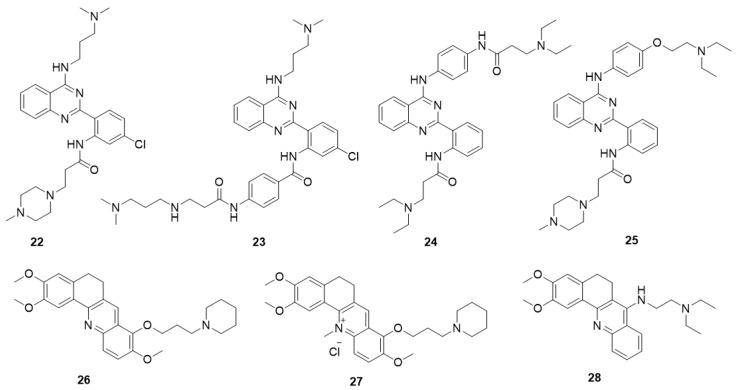
Structures of quinazoline derivatives and acridine derivatives.

The design of derivatives **22**–**25** is derived from the 11-substituted quindoline derivative SYUIQ-5 (Figure 5) [98,99,100]. By retaining the 11-substituted amino side chain of SYUIQ-5 and opening the five-membered indole ring, a non-coplanar flexible structure was obtained. This molecule has an “imitative” tetracyclic aromatic system formed via intramolecular hydrogen bonds, which enables the adoption of moderately twisted and near-planar conformations of the aryl groups and allows the ligand to stack well on the G-quartet. Therefore, these derivatives effectively bind to telomeric G-quadruplexes but not to duplexes. The most effective derivative, **22**, has a *K*_D_ value of 3.14 × 10^−7^ M. NMR and molecular docking analyses indicate that hydrogen bonding, π-π stacking interactions, and electrostatic interactions may account for the overall interaction. Derivative **22** also induced distinct cell senescence and telomere shortening in HL-60 cells at micromolar concentrations. An effective *N*-(2-(quinazolin-2-yl) phenyl)benzamide (QPB) derivative, **23**, was subsequently obtained. These derivatives have strong induction abilities, leading to the conversion of hybrid telomeric G-quadruplexes to parallel G-quadruplexes. SAR analysis indicates that the presence of an additional phenyl group and a chlorine substituent could greatly increase the stability and inducing ability of the derivative. Furthermore, a series of new 4-anilinoquinazoline derivatives were designed and synthetized; in these derivatives, the aromatic system of the quinazoline is expanded by introducing an anilino group at the 4-position of the quinazoline moiety to improve the G-quadruplex-binding activity and stability of the derivatives. The most effective derivative, **24**, has a *K*_D_ value of 2.1 × 10^−7^ M with the c-MYC G-quadruplex. Cellular studies showed that **24** significantly downregulated c-MYC gene transcription and expression in HeLa cells, presumably via the stabilization of the c-MYC G-quadruplex structure. In addition, real-time cell analyzer (RTCA) and colony formation assays indicated that **24** significantly inhibited HeLa cell proliferation but did not affect normal primary cultured mouse mesangial cells.

An in-house small-molecule library screening by SPR identified a quinazoline derivative, **25**, which showed a significant and specific interaction with the 5′-UTR IRES-A RNA G-quadruplex of the hVEGF-A mRNA and destabilized the G-quadruplex structure [101]. Competition dialysis measurements were performed to verify the selectivity of the compounds. The IRES-A (WT) oligomer and several other oligomers, including an RNA hairpin, random RNA, CT DNA, IRES-MU2, flanking sequences of IRES, DNA G-quadruplexes (TBA, c-KIT 1, and c-MYC Pu27), and other RNA G-quadruplexes (ADAM10, TERRA, BCL-2, and NRAS), were placed in independent dialysis cassettes after the general annealing process. The results showed that derivative **25** exhibited strongest binding with the IRES-WT and weaker binding with other DNA and RNA structures. Further cellular experiments revealed that derivative **25** downregulated hVEGF-A translation and significantly impeded tumor-cell migration. In vivo experiments showed that **25** exhibited tumor inhibition in MCF-7 xenograft tumors (Table 2). These findings provided a new strategy for the regulation of hVEGF-A translation, in which small molecules interact with the G-quadruplex structure in the 5′ UTR.

#### 5.5.2. Acridine Derivatives

Two series of 12-*N*-methylated and non-methylated 5,6-dihydrobenzo[*c*]acridine derivatives were designed by exploring the effect of the positive charge on berberine (Figure 5) [102,103]. The interactions of these derivatives with the c-MYC G-quadruplex were evaluated. Compared with the non-methylated derivative, **26**, the 12-*N*-methylated derivative, **27**, had stronger binding affinity and stabilizing ability toward the c-MYC G-quadruplex structure and could more effectively stack onto the G-quartet surface. All the derivatives had high selectivity for c-MYC G-quadruplex DNA over duplex DNA. The reverse transcription PCR (RT-PCR) assay showed that derivative **27** could downregulate transcription of the c-MYC gene in the Ramos cell line, containing an NHE III_1_ element, but had no effect in the CA46 cell line, in which the NHE III_1_ element had been removed. Recently, a series of 7-substituted 5,6-dihydrobenzo[*c*]acridine derivatives were synthesized as c-KIT G-quadruplex-binding ligands. These derivatives presented higher stabilization ability and binding affinity toward G-quadruplex DNA in the c-KIT oncogene promoter compared with that toward duplex DNA. The most active derivative, **28**, significantly reduced the transcription of c-KIT and exhibited a concomitant reduction of c-KIT protein levels in K562 cells. In addition, **28** induced apoptosis via suppression of Bcl-2, upregulation of Bax and activation of caspase-3.

#### 5.5.3. Multiaryl-Substituted Imidazole Derivatives 

**Figure 6:**
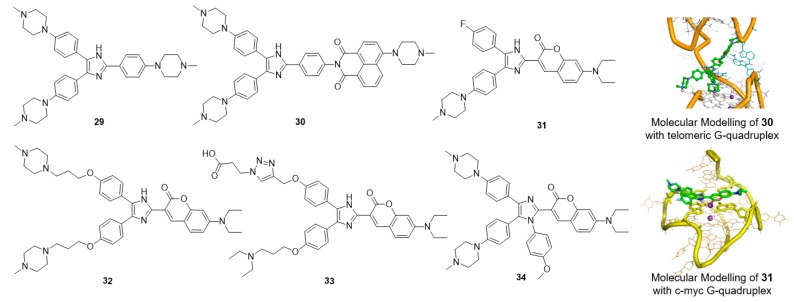
Multiaryl-substituted imidazole derivatives and binding mode with G-quadruplex.

Multiaryl-substituted imidazole derivatives designed for G-quadruplex binding were first discovered by using a 3D-QSAR pharmacophore model to screen our in-house derivative database (Figure 6) [62]. Derivative **29** exhibited potent binding and stabilization activity as well as 8.7-fold higher selectivity towards telomeric G-quadruplex DNA over duplex DNA. Inspired by these promising results, our research group further optimized this class of ligands using CADD methods in parallel with organic synthesis. Derivative **30**, with a 1,8-naphthalimide instead of an *N*-methyl piperazine group, was found to specifically bind to and strongly stabilize telomeric multimeric G-quadruplexes by intercalating into the pocket between the two quadruplex units. Further cellular studies indicated that **30** could induce telomeric DNA damage and telomere dysfunction, eventually leading to cell cycle arrest, apoptosis and senescence in SiHa cancer cells [50]. Such behavior differed from that of traditional telomeric G-quadruplex ligands, providing new insights for the development of selective anticancer drugs targeting telomeric multimeric G-quadruplexes. 

By replacing the *N*-methyl piperazine group with fluorophores, a series of small-molecular fluorescent probes were obtained. Derivative **31** exhibited distinct and specific fluorescence enhancement upon binding to parallel G-quadruplexes [104]. Derivative **32** greatly enhanced the detection limit of **31** for G-quadruplexes; the limit of detection (LOD) value of **32** for the G-quadruplex was up to 3 nM in solution and up to 5 ng in a gel matrix [105]. Derivative **33**, assembled with a triarylimidazole scaffold and a carboxyl side chain, was obtained as a positive hit by using in situ click chemistry, providing a method for identifying selective fluorescent probes for a specific topology of G-quadruplex nucleic acids. Derivative **34** showed great potential for the sensitive and selective detection of parallel G-quadruplexes [106]. A new tetra-arylimidazole probe, derivative **34**, exhibited significant and distinctive changes in both the absorption and fluorescence spectra in the presence of parallel G-quadruplexes but showed insignificant changes upon interaction with anti-parallel G-quadruplexes or non-quadruplex oligonucleotides. In view of this dual-output feature, **34** was further used to identify parallel G-quadruplexes from a large set of 314 oligonucleotides via a microplate reader, and thus, a high-throughput method for the characterization of parallel G-quadruplex topologies was established [107]. 

## 6. Summary and Outlook

Natural alkaloids and their heterocyclic derivatives are valuable assets for drug discovery and development. The polymorphism and structural complexity of natural alkaloids and their derivatives make it possible to discover potential anticancer drugs with high selectivity for G-quadruplexes over duplex DNA. In this review, we compiled the published information regarding natural alkaloids and heterocyclic derivatives that have been identified by our group as G-quadruplex ligands. The crescent-shaped planar chromophore moieties preferentially bind to and stabilize G-quadruplexes, while flexible side chains can increase the selectivity for G-quadruplexes over duplex DNA. The direction of the side chain also greatly affects the binding activity; derivatives with the side chain located outside the crescent ring system always shows good activity.

Over the past decade, our research group has developed a pool of ligands with diverse structures based on natural alkaloids and heterocyclic derivatives, as potential anticancer agents that target G-quadruplex structures. The activity data is summarized in Table 2 and Table 3. Although there is still a long way to go toward the improvement of cancer therapeutics, given the rapidly accumulating data on G-quadruplex structures and the biological functions and rapid development of G-quadruplex ligands, we are confident that a wealth of new derivatives that are less cytotoxic and have higher selectivity will emerge in the near future.

## Figures and Tables

**Table 1:** Methods for the discovery of G-quadruplex ligands.

Methods	Screening Index	Ref.
3D-QSAR Pharmacophore	Chemical feature of active compounds	[62]
Molecular docking	Estimated binding affinity to G-quadruplex	[63]
Fluorescence displacement (FID)	Competed binding towards G-quadruplex	[64]
NMR	Binding site with G-quadruplex	[65]
Fluorescence resonance energy transfer (FRET)	Stability effect of compounds on G-quadruplex	[66]
Surface plasmon resonance (SPR)	Binding affinity to G-quadruplex	[67]
G-quadruplex on Oligo Affinity Support (G4-OAS)	Binding affinity to G-quadruplex	[68]

**Table 2:** Anti-tumor activity of alkaloid derivatives in vivo.

Derivatives	Xenograft Model	Dosage (mg·kg^−1^)	Tumor Growth Inhibition	Days to Complete Response	Ref.
**9**	A549 human lung cancer	6.25; i.p.	38.1%	12	[77]
**10**	RAJI human Burkitt’s lymphoma	30; i.p.	27.4%	14	[78]
**15**	SiHa human cervical squamous cancer	20; i.p.	35.2%	20	[79]
**16**		5; i.p.	64.8%	21	[79]
**21**		1; i.p.	49.3%	20	[79]
**24**	MCF-7 human breast cancer	7.5; i.p.	60.1%	20	[79]

**Table 3:** Biological activity of alkaloid derivatives in the above sections.

Compounds		Target G4	Biochemical Activity			Cytotoxicity (IC_50_)	Ref.
			Δ*T*_m_	*K*_D_	^tel^IC_50_		
**Quindoline derivatives**	**3**	telomere	n.d.	n.d.	16 μM ^b^	6.3 μM (SKOV-6)	[69]
	**4**	telomere	13.0 °C	n.d.	6.4 μM ^b^	n.d.	[70]
	**5**	telomere	21.0 °C	n.d.	0.44 μM ^b^	1.68 μM (SW620)	[73]
	**6**	telomere	14.0 °C	n.d.	0.31 μM ^b^	1.21 μM (HL-60)	[74]
	**7**	telomere	24.3 °C	0.19 μM (SPR)	5.5 μM ^c^	45 μM (HL-60)	[75]
	**9**	c-MYC	13.9 °C	1.1 μM (MST)	n.d.	0.19 μM (RAJI)	[77]
	**10**	c-MYC	27.0 °C	1.3 μM (MST)	n.d.	4.7 μM (RAJI)	[78]
**Isaindigotone derivatives**	**12**	telomere	21.9 °C	0.1 μM (FIT)	7.8 μM ^c^	29 μM (CA46)	[81,82,83]
	**13**	c-KIT	13.6 °C	0.6 μM (SPR)	n.d.	6.8 μM (HGC-27)	[81,82,83]
	**14**	c-MYC ^a^	n.d.	5.29 μM (SPR)	n.d.	13.1 μM (Hela)	[79]
	**15**	c-MYC ^a^	12.1 °C	17.0 μM (MST)	n.d.	16.0 μM (SiHA)	[79]
	**16**	c-MYC ^a^	0.5 °C	2.0 μM (MST)	n.d.	1.78 μM (SiHA)	[79]
**Berberine derivatives**	**18**	telomere	23.4 °C	n.d.	n.d.	n.d.	[95]
	**19**	telomere	28.2 °C	0.3 μM (FIT)	16 μM ^b^	n.d.	[95]
	**20**	telomere	25.0 °C	0.1 μM (FIT)	14 μM	n.d.	[95]
	**21**	telomere	9.0 °C	14.8 μM	n.d.	1.7 μM (HL-60)	[95]
**BRACO-19**		telomere	27.5 °C	0.032 μM (SPR)	0.11 μM ^c^	8 μM (21NT)	[19]
**Telomestatin**		telomere	n.d.	n.d.	0.005 μM ^b^	0.8 μM (LAN1)	[108]

^a^ target c-MYC G-quadruplex by interacting with NM23-H2; ^b^ telomerase inhibition by TRAP assay; ^c^ telomerase inhibition by TRAP-LIG assay.
